# Blockade of Hedgehog Signaling Synergistically Increases Sensitivity to Epidermal Growth Factor Receptor Tyrosine Kinase Inhibitors in Non-Small-Cell Lung Cancer Cell Lines

**DOI:** 10.1371/journal.pone.0149370

**Published:** 2016-03-04

**Authors:** Xiao-Yan Bai, Xu-Chao Zhang, Su-Qing Yang, She-Juan An, Zhi-Hong Chen, Jian Su, Zhi Xie, Lan-Ying Gou, Yi-Long Wu

**Affiliations:** 1 Department of Pulmonary Oncology, Guangdong Lung Cancer Institute, Guangdong General Hospital & Guangdong Academy of Medical Science, Guangzhou 510080, China; 2 Southern Medical University, Guangzhou 510515, China; Indiana University School of Medicine, UNITED STATES

## Abstract

Aberrant activation of the hedgehog (Hh) signaling pathway has been implicated in the epithelial-to-mesenchymal transition (EMT) and cancer stem-like cell (CSC) maintenance; both processes can result in tumor progression and treatment resistance in several types of human cancer. Hh cooperates with the epidermal growth factor receptor (EGFR) signaling pathway in embryogenesis. We found that the Hh signaling pathway was silenced in EGFR-TKI-sensitive non-small-cell lung cancer (NSCLC) cells, while it was inappropriately activated in EGFR-TKI-resistant NSCLC cells, accompanied by EMT induction and ABCG2 overexpression. Upregulation of Hh signaling through extrinsic SHH exposure downregulated E-cadherin expression and elevated Snail and ABCG2 expression, resulting in gefitinib tolerance (*P < 0*.*001*) in EGFR-TKI-sensitive cells. Blockade of the Hh signaling pathway using the SMO antagonist SANT-1 restored E-cadherin expression and downregulate Snail and ABCG2 in EGFR-TKI-resistant cells. A combination of SANT-1 and gefitinib markedly inhibited tumorigenesis and proliferation in EGFR-TKI-resistant cells (*P < 0*.*001*). These findings indicate that hyperactivity of Hh signaling resulted in EGFR-TKI resistance, by EMT introduction and ABCG2 upregulation, and blockade of Hh signaling synergistically increased sensitivity to EGFR-TKIs in primary and secondary resistant NSCLC cells. E-cadherin expression may be a potential biomarker of the suitability of the combined application of an Hh inhibitor and EGFR-TKIs in EGFR-TKI-resistant NSCLCs.

## Introduction

Lung cancer is a leading cause of cancer death worldwide, and poses a significant risk to human health. Median survival of patients with advanced non-small-cell lung cancer (NSCLC) who receive standard chemotherapy is only 9–12 months[1]. The advent of molecularly targeted therapies has shed much light on lung cancer treatment. Epidermal growth factor receptor tyrosine kinase inhibitors (EGFR-TKIs) have become the standard first-line treatment for advanced NSCLC with sensitive EGFR mutations[2, 3]. However, almost all patients inevitably develop resistance within 6–12 months after the initiation of EGFR-TKI treatment[4]. Although several mechanisms, including the T790M secondary mutation[5, 6], MET amplification[7, 8], hepatocyte growth factor (HGF) overexpression[9], and the KRAS mutation[10, 11], have been reported, overcoming EGFR-TKI resistance remains a challenge in clinical practice.

Emerging evidence suggests that induction of the epithelial-to-mesenchymal transition (EMT) in malignancy results in tumor progression and drug resistance[12, 13]. Cancer stem-like cells (CSCs) are another reason for resistance to conventional tumor therapy[14]. Recent reports indicated that EMT processes could generate CSCs[15]. The hedgehog (Hh) signaling pathway, a major regulator of many fundamental processes, closely regulates cell proliferation, differentiation, EMT, and stem cell maintenance in vertebrate embryonic development, and aberrant Hh activation in adult tissues has been implicated in tumorigenesis, self-renewal, and drug resistance in several types of human cancer, including lung cancer[16–18]. Three Hh homologs have been identified in humans: Sonic hedgehog (SHH), Indian hedgehog (IHH), and Desert hedgehog (DHH)[19, 20]. The Hh signaling pathway is initiated by the Hh ligand binding to a 12-transmembrane protein, “patched” (PTCH). In the absence of the Hh ligand, PTCH represses the activity of Smoothened (SMO), a G-like protein-coupled receptor, by preventing its localization to the primary cilium. Once Hh binds to PTCH, the inhibition of SMO is relieved, allowing SMO to translocate to the primary cilium and to transduce the Hh signal to the GLI family of zinc-finger transcription factors (GLI1, GLI2, GLI3)[20, 21]. The GLIs then translocate into the nucleus to regulate the activation of Hh target genes involved in various processes, such as feedback regulation, proliferation, EMT, and self-renewal[22, 23].

During embryogenesis, the Hh signaling pathway cooperates with the EGFR signaling pathway in the process of neocortical stem cell proliferation[23]. Accumulating evidence indicates that cooperative interactions between the Hh and EGFR pathways result in synergistic regulation of GLI target gene expression and contribute to the malignant transformation of cancer cells, *in vitro* and *in vivo*[24, 25]. Taken together, we supposed that stimulation of the Hh signaling pathway may bypass or attenuate the therapeutic efficacy of EGFR-TKIs in NSCLC. Thus, we used EGFR-TKI-sensitive and -resistant NSCLC cell models to first demonstrate that upregulation of Hh signaling contributed to EGFR-TKI-resistance. We found that the combination of an Hh signaling inhibitor and EGRF-TKIs had a marked synergistic effect in NSCLC cells.

## Materials and Methods

### Antibodies and reagents

Gefitinib was provided by AstraZeneca (Cheshire, UK). The Hh inhibitor SANT-1 was purchased from TOCRIS Bioscience. Recombinant human Shh N-terminus was purchased from R&D Systems. Antibodies against Snail (#9585), E-cadherin (#3195), and ABCG2 (#4477) were obtained from Cell Signaling Technology. Antibodies against GLI1 (ab92611) were purchased from Abcam.

### Cell lines

Human bronchial epithelial cell line (HBE) was obtained from Sciencell Company and grown in bronchial epithelial cell medium (Sciencell, 3211). Human lung adenocarcinoma A549 cells were obtained from the American Type Culture Collection (Manassas, VA) and maintained in our laboratory. Human lung adenocarcinoma H1975 and PC9 cells were obtained from the American Type Culture Collection. They were kindly provided by Dr. Tony Mork (Chinese University of Hong Kong). A549 cells are an EGFR-TKI primary resistant cell line due to their harboring K-ras G12S mutation. H1975 cells are an EGFR-TKIs secondary resistant cell line due to the EGFR L858R and T790M mutations. PC9 cells harbor the EGFR exon 19 frame deletion and are highly sensitive to EGFR-TKIs. NSCLC cell lines were maintained in RPMI 1640 medium supplemented with 10% fetal bovine serum, penicillin (100 UI/mL), and streptomycin (100 μg/mL). All cells were cultured at 37°C in a humidified atmosphere with 5% CO_2_.

### Patients

NSCLC tumor specimens containing the EGFR exon 19 deletion or L858R sensitive mutation, EGFR T790M secondary mutation, and KRAS mutation were obtained from Guangdong General Hospital (Guangzhou, China) under institutional review board approval. This study was approved by the Ethics Committee of Guangdong General Hospital (YUE medical ethics no. 2013185 (R2)). All patients provided written informed consent. The presence of EGFR and KRAS mutations in each specimen was determined by PCR-based direct sequencing.

### Immunocytochemistry and immunohistochemistry

NSCLC cells were fixed and permeabilized with cold acetone; frozen sections (5 μm) were fixed in cold methanol for 10 min and air-dried. These slides were immersed in 3% H_2_O_2_ for 10 min to block endogenous peroxidases, then incubated with 10% goat serum albumin for 10 min at room temperature to block non-specific antibody binding. Subsequently, cells were stained with GLI1, E-cadherin, Snail, and ABCG2 primary antibodies (1:100) overnight at 4°C. After washing with PBS, cells were incubated with Envision^+^/HRP against rabbit (DAKO GK400305, Glostrup, Denmark) for 30 min, followed by 3,3’-diaminobenzidine (DAB) detection. Slides were counterstained with hematoxylin and after dehydration were mounted permanently. Negative controls were performed in all cases by omitting the primary antibodies.

All slides were evaluated independently by two pathologists who were blinded to the case information. Cases with staining in > 10% of cells were considered positive. Immunohistochemical reactivity was graded on a scale of 0 to 3 according to staining intensity and the percentage of immunopositive cells, as follows: 0, no staining, < 10% positive cells, 1, weak staining in > 10% of tumor cells or moderate staining in 10–40% of tumor cells, 2, moderate staining in > 40% of tumor cells or strong staining in 10–40% of tumor cells, or 3, strong staining in > 40% of tumor cells[26].

### RNA isolation and quantitative real-time PCR analysis

Total RNA was extracted using TRIZOL reagent (Invitrogen, Carlsbad, CA, USA) from the cell lines according to the manufacturer’s instructions. Total RNA was quantified using the ultraviolet spectrophotometer (Nanodrop ND-1000). RNA integrity was assessed using 1% denaturing agarose gel electrophoresis. The cDNA synthesis was performed using high capacity cDNA reverse transcription kit (ABI, USA) according to the manufacturer’s instructions. Q-PCR reactions were performed using TaqMan gene expression master mix (ABI, USA), β-ACTIN was used as an endogenous control to normalize the data. All qPCR reactions were performed in triplicate. Primer sequences used in this study were the following: 5’-AGCGTGAGCCTGAATCTGTG-3’ (forward) and 5’-CAGCATGTACTGGGCTTTGAA-3’ (reverse) for GLI1, 5’-AAAGACCTGTACGCCAACAC-3’ (forward) and 5’-GTCATACTCCTGCTTGCTGAT-3’ (reverse) forβ-ACTIN. The relative RNA expression level of GLI1 was calculated with 2^-Δct^ methods.

### Western blot analysis

Cell were cultured in 25cm^2^ culture bottles and harvested in the log-growth phase for protein extraction. Cell Total protein was extracted from treated cells using radioimmunoprecipitation assay (‘RIPA’) buffer, supplemented with protease inhibitors PMSF. The protein concentration of each lysate was determined using the bicinchoninic acid assay. Proteins(30ug/well) were separated by SDS-PAGE and transferred onto PVDF membranes. The proteins were blocked by 5% Proteins non-fat milk for 1h at room temperature. Proteins were detected by incubation of the membranes in the presence of the following primary antibodies: GAPDH (1:1000), ABCG2 (1:500), Snail (1:500), E-cadherin (1:500) and Gli1 (1:500) and then incubated with horseradish peroxidase-conjugated secondary antibody at room temperature for 1h. Antibody binding was detected by an enhanced chemiluminescence kit (Thermo, Rockford, IL, USA).

Films were scanned and analyzed with image J software for protein quantification. The relative protein levels were counted using a comparison to untreated control.

### Cell proliferation assay

Cells were seeded in 96-well plates at a density of 2,000 /well for PC9, 3,000 /well for H1975, and 1,500 /well for A549. Following overnight attachment, cells were treated, with five replicates, with SHH-N 1 μg/mL (R&D Systems) for 24 h, followed by various concentrations of gefitinib for 72 h, or treated directly with various concentrations of inhibitors for 72 h. The number of viable cells was assessed in the five replicate wells per assay condition using the MTT assay (Sigma) according to the manufacturer’s instructions. Each experiment was repeated at least three times independently.

### Clonogenic assay

Cells were seeded in six-well plates at a density of 1×10^2^ /well. Following overnight attachment, cells were treated, in triplicate, with the drug on days 0, 3, and 6. After incubation for 10 days at 37°C in a humidified atmosphere with 5% CO_2_, cells were fixed with methanol and acetic acid (3:1, v/v). Next, colonies were stained with 0.5% crystal violet and quantified by directly counting colonies. The experiment was repeated at least three times independently.

### Statistical analyses

Statistical analyses were performed using the SPSS software (ver. 13.0; SPSS Inc., Chicago, IL). Results are presented as means ± SE of at least three experiments. First, data were tested for a normal distribution and homoscedasticity. Differences in Q-PCR, western blot gray scale values, and cell clone formation, between groups were assessed by one-way ANOVA, differences in cell proliferation were tested by a factorial analysis, and intragroup differences were evaluated using the LSD test if the data were normally distributed and showed homoscedasticity. Otherwise, a Welch test was used, and intragroup differences were evaluated using Dunnett’s T3 test. Differences in ranked data among groups were assessed using a Kruskal-Wallis H-test. Correlations of ranked data were analyzed using Spearman’s rank correlation test. *P* values < 0.05 were considered to indicate statistical significance in all cases.

## Results

### Differences in Hh signaling pathway activity between EGFR-TKI-sensitive and -resistant NSCLC cells

**Fig 1:**
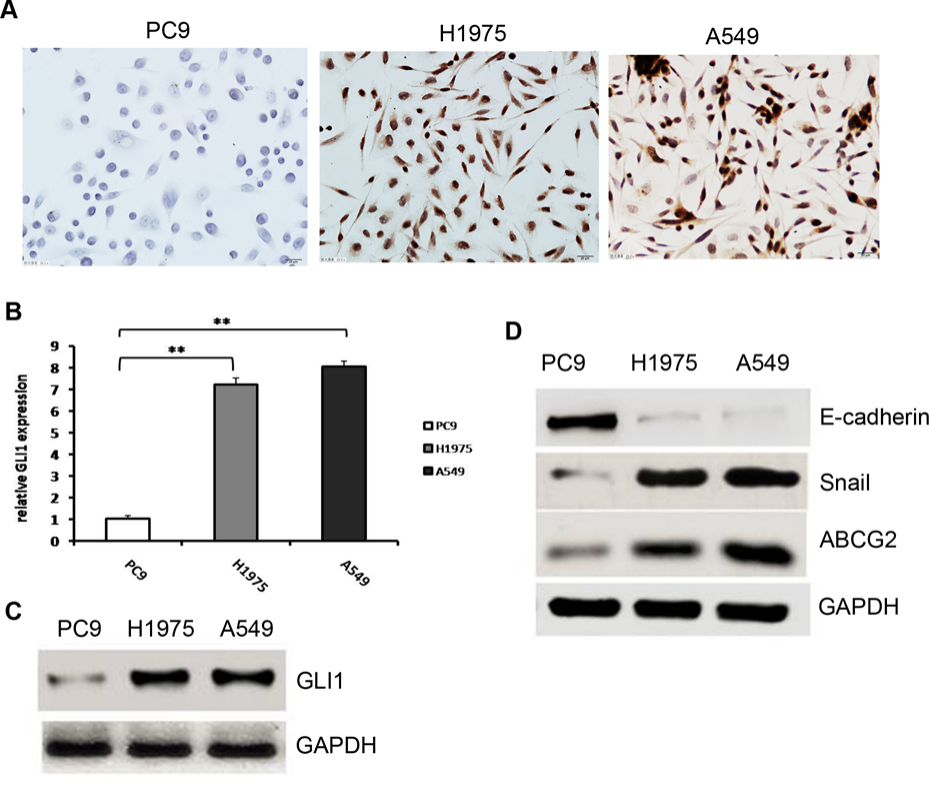
Hh pathway activity in EGFR-TKI-sensitive and -resistant cells. (A). Differences in GLI1 expression in EGFR-TKI-sensitive and -resistant NSCLC cells determined with immunocytochemistry. GLI1 was expressed in the nuclei of EGFR-TKI-resistant cell lines A549 and H1975, but GLI1 expression was negative in the EGFR-TKI-sensitive cell line PC9. (B). Relative GLI1 mRNA expression in EGFR-TKI-sensitive and -resistant NSCLC cells determined by Q-PCR; The expression of PC9 was significantly lower than that of H1975 and A549; ** (*P*<0.01). (C). Differences in GLI1 expression in EGFR-TKI-sensitive and -resistant NSCLC cells determined by western blot. (D) Differences in E-cadherin, Snail, and ABCG2 expression in EGFR-TKI-sensitive and -resistant NSCLC cells determined by western blot.

To assess Hh signaling pathway differences between EGFR-TKI-sensitive and -resistant NSCLC cells, three NSCLC cell lines, PC9, H1975, and A549, harboring different mutations and differing in sensitivity to TKIs, were used. First, expression of GLI1, a marker of activation of the Hh signaling pathway, was determined by immunocytochemistry. As shown in Fig 1A, GLI1 was expressed in the nuclei of EGFR-TKI-resistant cell lines A549 and H1975, but GLI1 expression was negative in the EGFR-TKI-sensitive cell line PC9. We confirmed this result by Q-PCR and Western blot analysis. As shown in Fig 1B and 1C, GLI1 was expressed at a very low level in PC9 compared with H1975 and A549 cells *(P>0*.*001* and *P = 0*.*005* respectively). Previous studies indicated that Hh signaling regulates EMT via upregulation of the transcription factor Snail and downregulation of E-cadherin[27, 28]. The stem cell marker ABCG2 is also a direct target of the Hh signaling pathway[29]. To further clarify the Hh pathway differences between EGFR-TKI-sensitive and -resistant cells, these three important downstream target genes were examined by Western blotting. We found that Snail expression was considerably weaker in the EGFR-TKI-sensitive PC9 cell line compared with the EGFR-TKI-resistant cell lines H1975 and A549 (*P* = 0.001). E-cadherin expression in PC9 cells was quite high, while its expression was very weak in the EGFR-TKI-resistant cell lines H1975 and A549 (*P*<0.001; Fig 1D). This result was consistent with previous reports that Snail expression was inversely correlated with that of E-cadherin[30, 31]. ABCG2 expression was also quite high in the EGFR-TKI-resistant cell lines H1975 and A549 compare with its expression in the EGFR-TKI-sensitive PC9 cell line (*P*<0.001; Fig 1D). Together, these results indicated a significant difference in the activity of the Hh signaling pathway between EGFR-TKI-sensitive and -resistant cells. The Hh signaling pathway was aberrantly activated in EGFR-TKI-resistant cells, while it was silenced in EGFR-TKI-sensitive cells.

### Upregulation of Hh signaling pathway activity by exposure to SHH lead to gefitinib tolerance accompanied by EMT induction and ABCG2 upregulation in EGFR-TKI-sensitive NSCLC cells

**Fig 2:**
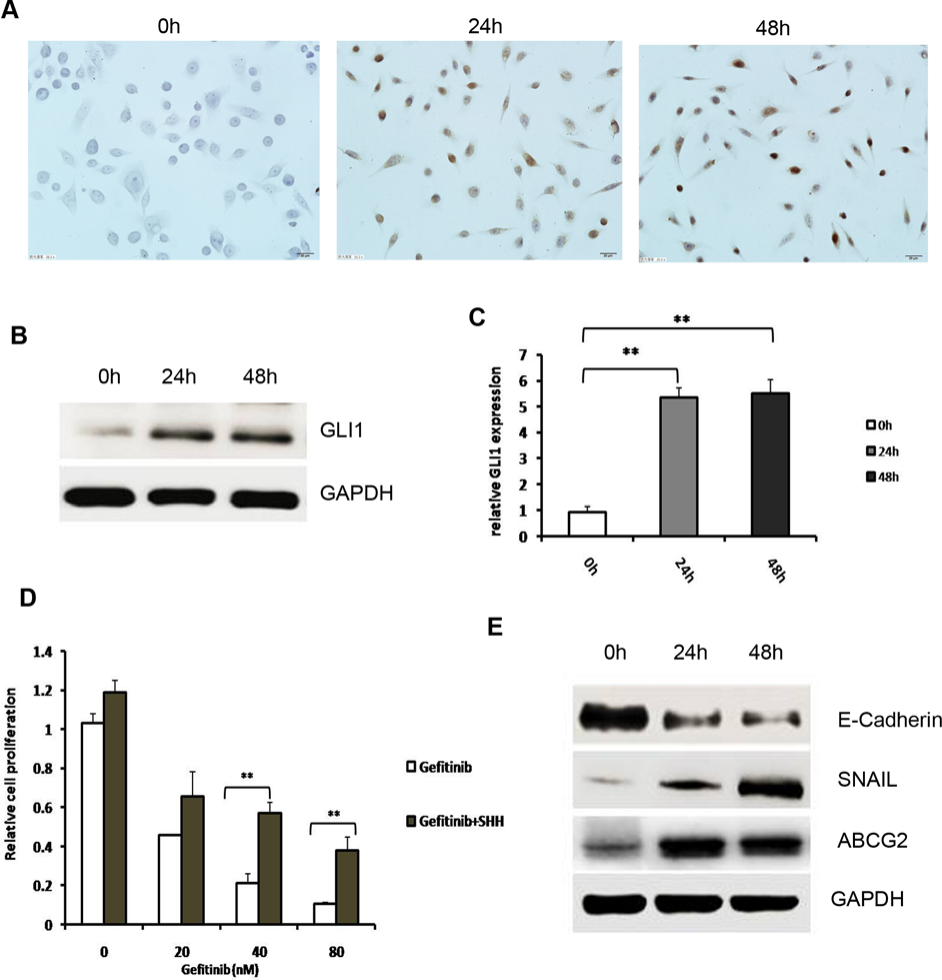
Upregulation of Hh signaling pathway activity by exposure to SHH lead to gefitinib tolerance accompanied by EMT induction and ABCG2 upregulation in EGFR-TKI-sensitive NSCLC cells. (A) Immunocytochemical analysis of GLI1 in PC9 cells after N-Shh (0.5 μg/mL) treatment for 0, 24, and 48h. GLI1 expression in PC9 cells was negative without exposure to N-Shh. GLI1 was expressed in the nuclei after N-Shh treatment for 24 and 48 h. (B) Western blot analysis of GLI1 in PC9 cells after N-Shh (0.5 μg/mL) treatment for 0, 24, and 48h. (C) Q-PCR analysis of GLI1 mRNA relative expression in PC9 cells after N-Shh (0.5 μg/mL) treatment for 0, 24, and 48h; GLI1 expression was obviously elevated after exposure to N-Shh for 24 h and 48 h; ** (*P*<0.01). (D) Proliferation of PC9 cells was assessed by MTT assay after treatment with the indicated concentrations of gefitinib with or without pre-exposure to extrinsic N-Shh (0.5 μg/mL) for 24h; compared with the gefitinib single-agent group, exposure to extrinsic N-Shh significantly increased gefitinib tolerance in PC9 cells ** (*P*<0.01). (E) Western blot analysis of E-Cadherin, Snail and ABCG2 in PC9 cells after N-Shh (0.5 μg/mL) treatment for 0, 24, and 48h.

Based on the results above, we hypothesized that aberrant activation of the Hh signaling pathway may contribute to the EGFR-TKI resistance in NSCLC by affecting EMT and CSC maintenance. To examine this possibility, we tested the role of Shh signaling in NSCLC cells. First, EGFR-TKI-sensitive PC9 cells were treated with N-Shh (0.5 μg/mL). Immunocytochemistry result showed that GLI1 expression in PC9 cells was negative without exposure to N-Shh, but it was expressed in the nuclei after N-Shh treatment for 24 and 48 h (Fig 2A). Western blotting and Q-PCR results showed that GLI1 expression was obviously elevated after exposure to N-Shh for 24 h and 48 h (*P* = 0.008 and *P*<0.001 respectively; Fig 2B and 2C). These results demonstrated that Hh signaling was activated by extrinsic N-Shh in PC9 cells.

Next, EGFR-TKI-sensitive cells PC9 were treated with increasing concentrations of gefitinib with or without exposure to extrinsic N-Shh (0.5 μg/mL), then their viability was assessed. Our results showed that, compared with the gefitinib single-agent group, exposure to extrinsic N-Shh significantly increased gefitinib tolerance in PC9 cells (*P* = 0.001; Fig 2D and S1 and S2 Tables). These findings show that aberrant activation of the Shh signaling pathway leads to EGFR-TKI resistance in NSCLC cells.

To examine the molecular mechanisms underlying the contribution of Shh signaling to EGFR-TKI resistance in NSCLC cells, we examined Snail, E-cadherin, and ABCG2 expression at 0, 24, and 48 h after treatment of PC9 cells with N-Shh (0.5 μg/mL) by Western blotting. As shown in Fig 2E, after exposure to N-Shh for 24 h, the expression of Snail was elevated (*P*<0.001), the expression of E-cadherin was obviously attenuated (*P* = 0.003), and ABCG2 expression was markedly upregulated in PC9 cells (*P* = 0.008). These effects were sustained for 48 h following N-Shh stimulation. These results confirmed that hyperactivation of Hh signaling contributed to EGFR-TKI resistance in NSCLC cells through activation of the EMT transition and the ABCG2 upregulation.

### Hh inhibition reversed EMT induction and decreased ABCG2 expression in EGFR-TKI-resistant NSCLC cells

**Fig 3:**
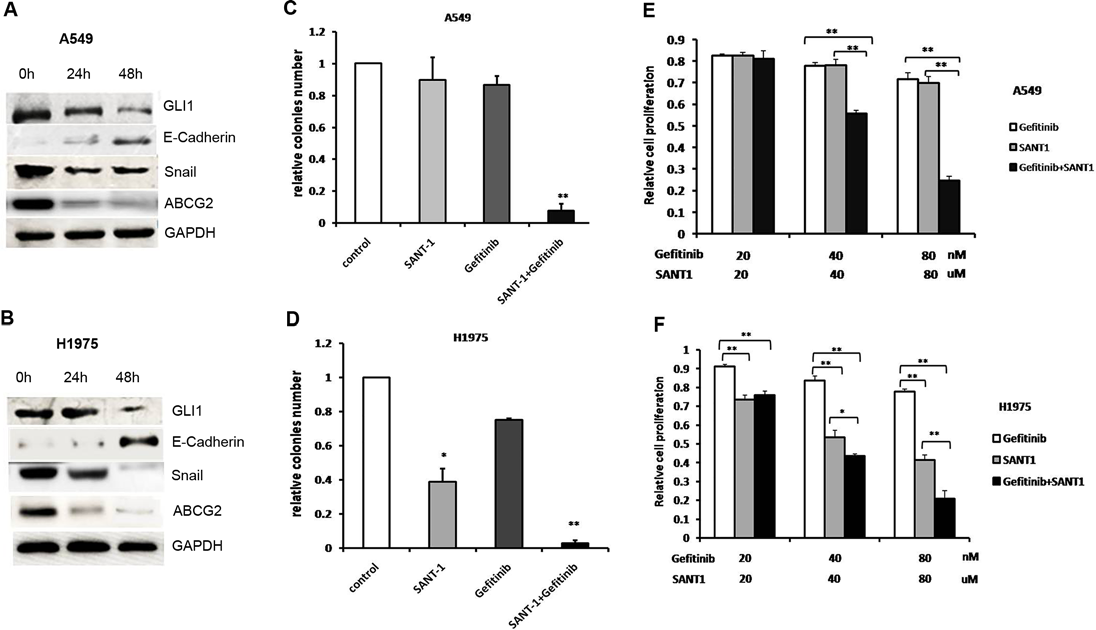
Hh inhibition reversed EMT and decreased the ABCG2 expression in EGFR-TKI-resistant NSCLC cells and the combination of gefitinib and SANT1 synergistically inhibited tumorigenesis and proliferation in EGFR-TKI-resistant NSCLC cell lines. (A) and (B), Western blot analysis of the Hh signaling pathway activity and its target genes E-Cadherin, Snail and ABCG2 in EGFR-resistant cells A549 and H1975 after SANT-1 treatment for 0, 24, and 48 h. (C) and (D), colony formation quantitative analysis in A549 and H1975 cells; gefitinib alone or SANT1 alone did not inhibit clonogenic growth effectively. However, colonies very barely formed in the group subjected to combined SANT-1 and gefitinib treatment in A549 and H1975 cells; * (*P*<0.05), ** (*P*<0.01). (E) and (F), Proliferation of A549 and H1975 cells was assessed by MTT assay after treatment with the indicated concentrations of gefitinib alone, SANT-1 alone, or the combination of gefitinib and SANT-1; * (*P<0*.*05)*, **(*P<0*.*01*).

Next, to further assess the molecular mechanisms of Hh signaling in EGFR-TKI-resistant NSCLC cells, we examined GLI1, Snail, E-cadherin, and ABCG2 expression at 0, 24, and 48 h after treatment of the EGFR-TKI-resistant cell lines H1975 and A549 with SANT-1 (40 μM). The results indicated that after treatment with SANT-1 for 24 h, GLI1 expression was downregulated (*P*<0.001) and Snail expression was significantly weakened in both cell lines (*P*<0.001 and *P* = 0.003 respectively); after treatment with SANT-1 for 48 h, Snail expression was almost absent in H1975 cells (Fig 3A and 3B). Conversely, E-cadherin expression was elevated significantly following treatment of EGFR-TKI-resistant cell lines with SANT-1 for 48 h (*P*<0.001). ABCG2 expression was negligible after SANT-1 treatment for 24 h (Fig 3A and 3B). These findings demonstrated that Hh inhibition reversed EMT and decreased the ABCG2 expression in EGFR-TKI-resistant NSCLC cells.

### The combination of gefitinib and SANT-1 synergistically inhibited tumorigenesis and proliferation in EGFR-TKI-resistant NSCLC cell lines

To further demonstrate the role of the Hh signaling pathway in NSCLC cells, we blocked Hh signaling using the SMO inhibitor SANT-1. The IC_50_ of SANT-1 in most NSCLC cell lines is ~40 μM[18], and the IC_50_ of gefitinib in EGFR-TKI-sensitive cells is ~20 nM[32]. Thus, the EGFR-TKI-resistant cell lines A549 and H1975were treated with 40 μM SANT-1 alone, 20 nM gefitinib alone, or a combination of SANT-1 and gefitinib. As shown in Fig 3C and 3D, SANT-1 and gefitinib alone did not inhibit clonogenic growth (*P* = 0.252 and *P* = 0.187 respectively). However, colonies very barely formed in the group subjected to combined SANT-1 and gefitinib treatment (*P* < 0.001). These results indicate that SANT-1 and gefitinib may have a synergistic effect in EGFR-TKI-resistant NSCLC cells. To confirm this, we treated the EGFR-TKI-resistant NSCLC cell lines A549 and H1975 with increasing concentrations of SANT-1 alone, gefitinib alone, and combinations of SANT-1 and gefitinib, and then assessed their proliferation. The results showed that A549 cells were resistant not only to gefitinib but also to SANT-1 (*P* = 0.503; Fig 3E and S3 and S4 Tables). This result is consistent with a previous report that A549 cells showed hyperactivation of Hh signaling, but were resistant to Hh-signaling inhibitors [18]. However, the combination of gefitinib and SANT-1 inhibited the proliferation of A549 cells (*P <* 0.001; Fig 3E and S3 and S4 Tables). Although, compared with gefitinib, SANT-1 was more effective in H1975 cells (*P* = 0.002), H1975 cells showed the ‘best’ response to the combination of gefitinib + SANT1 (*P <* 0.001; Fig 3F and S5 and S6 Tables). Taken together, these results confirmed that the combination of an Hh signaling inhibitor and EGFR-TKIs had marked synergistic effects on EGFR-TKI-resistant NSCLC cells.

### Activity of the Hh signaling pathway in EGFR-TKI-sensitive and -resistant NSCLC tissues

**Fig 4:**
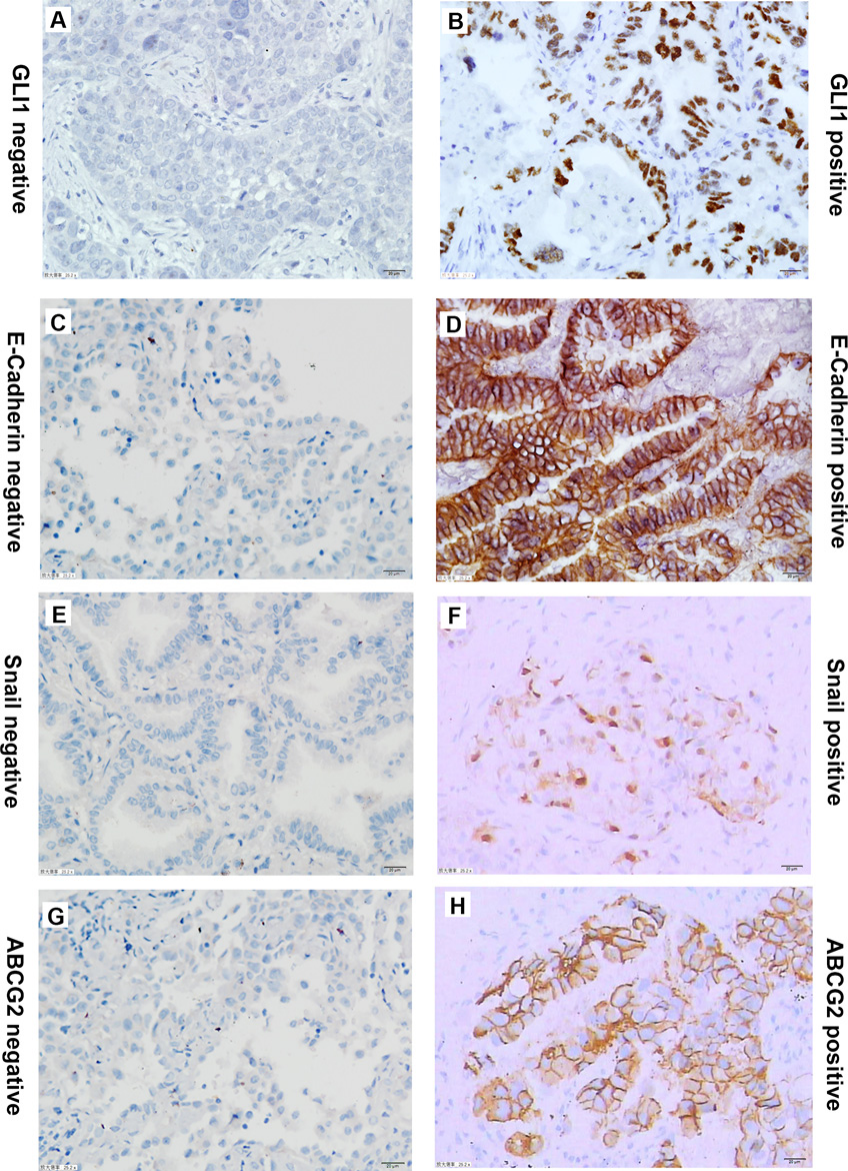
Representative immunostaining of GLI1, ABCG2, E-Cadherin and Snail in NSCLC tissues. (A) and (B), negative control and positive GLI1 expression in NSCLC tissues. (C) and (D), negative control and positive E-Cadherin expression in NSCLC tissues. (E) and (F), negative control and positive Snail expression in NSCLC tissues.(G) and (H), negative and positive ABCG2 expression in NSCLC tissues.

To further understand the difference in Hh signaling pathway activity between EGFR-TKI-sensitive and -resistant NSCLCs, expression of GLI1, ABCG2, Snail, and E-cadherin was assessed by IHC in four NSCLC tissues that contained the EGFR exon 19 deletion or the L858R sensitive mutation, four tissues that contained the EGFR T790M secondary mutation, and four tissues that contained the KRAS mutation (Fig 4). The results indicated that GLI1 was expressed in both EGFR-TKI-sensitive and -resistant tissues. Because of the small size of the samples, GLI1 expression showed no statistically significant difference among the three groups (*P* = 0.108). However, the mean rank of EGFR-TKI-sensitive tissues was 5.25, that of secondary resistant mutation tissues was 4.88, and the mean rank of KRAS mutation tissues was 9.38. Thus, a trend towards higher GLI1 expression was found in tissues that contained the KRAS mutation (Tables 1 and 2). KRAS mutation-harboring NSCLCs may have higher Hh signaling pathway activity compared with those with EGFR-TKI-sensitive mutations and secondary resistant mutations.

**Table 1:** Expression of GLI1, ABCG2, E-cadherin and Snail in NSCLC tissues tested by immunohistochemistry.

ID	Gender	Age	Gene statues	EGFR-TKI	GLI1	ABCG2	E-cadherin	Snail
L1756	F	64	DEL	Sensitive	0	0	3	0
L2365	M	59	DEL	Sensitive	1	1	3	0
L2186	F	63	DEL	Sensitive	2	0	1	1
L2160	F	50	L858R	Sensitive	0	0	3	0
L1433	M	45	T790M+del	Resistance	2	1	2	1
L1439	M	56	T790M	Resistance	1	0	0	1
L0353	M	43	T790M+del	Resistance	1	2	3	0
L1423	F	53	T790M+L858R	Resistance	0	0	2	0
L1301	M	66	KRAS	Resistance	2	2	2	2
L1677	M	61	KRAS	Resistance	3	0	0	0
L1834	M	63	KRAS	Resistance	2	2	3	0
L0357	F	58	KRAS	Resistance	2	2	2	1

**Table 2:** Expression differences of GLI1, ABCG2, E-cadherin and Snail in EGFR-TKIs sensitive and resistant tissues.

Protein	EGFR-TKI	Mean rank	*χ*^*2*^-value	*P*-value
GLI1	Sensitive	5.25	4.460	0.108
	Secondary resistance	4.88		
	Primary resistance	9.38		
ABCG2	Sensitive	5.13	2.155	0.340
	Secondary resistance	6.00		
	Primary resistance	8.38		
E-cadherin	Sensitive	8.00	1.179	0.555
	Secondary resistance	5.75		
	Primary resistance	5.75		
Snail	Sensitive	5.38	0.838	0.658
	Secondary resistance	6.75		
	Primary resistance	7.38		

ABCG2 expression did not differ significantly among EGFR-TKI-sensitive, secondary resistant, and primary resistant NSCLC tissues (*P* = 0.340). E-cadherin was moderately or strongly expressed in most NSCLC tissues (9/12, *P* = 0.555). Conversely, Snail was negative or weakly expressed in most tissues (11/12, *P* = 0.658) (Tables 1 and 2).

Correlation analysis showed that E-cadherin expression was significantly negatively correlated with Snail expression (*r* = 0.582, *P* = 0.047). This result was consistent with the cell experiments. Additionally, GLI1 expression was negatively correlated with E-cadherin expression (*r* = 0.408, *P* = 0.188), and positively correlated with Snail (*r* = 0.372, *P* = 0.300) and ABCG2 (*r* = 0.386, *P* = 0.215) expression. Although the correlations lacked statistical significance because of the small sample size, the tendency was consistent with the cell experiments.

## Discussion

The Hh signaling pathway is an effective therapeutic target in the treatment and prevention of many types of human cancer. Inappropriate activation of the Hh signaling pathway has been reported in NSCLC[17, 18, 33, 34]. Previous studies have shown that EMT induction is associated with sensitivity to EGFR-TKI in lung cancer[35–37]. The hedgehog signaling pathway tightly regulates EMT induction through its downstream target genes, such as Snail, ZEB1, and TWIST2[22]. Downregulation or loss of E-cadherin expression is a hallmark of EMT in embryonic development and cancer progression. Snail expression is inversely correlated with E-cadherin in epithelial tumor cell lines[30, 31]. ABCG2 is one of the major multidrug resistance (MDR) pumps; it is also a stem cell marker and is closely associated with resistance to several drugs[38, 39]. The Hh signaling pathway directly regulates the activity of ABCG2[29]. Hh antagonists can inhibit the activity of ABCG2 and resensitize NSCLC cells to mitoxantrone and topotecan *in vitro*[40]. Hh and EGFR signaling cooperatively regulate stem cell proliferation in the postnatal and adult brain[23, 41]. Simultaneous blockade of Hh and EGFR signaling inhibited proliferation and induced apoptosis, and improved the cytotoxic effects of docetaxel in metastatic prostate cancer cells[42]. Given this background, we hypothesized that the Hh signaling pathway might contribute to EGFR-TKI resistance in NSCLC by disregulated the EMT and ABCG2 activity.

To clarify the connection between Hh signaling and EGFR-TKI resistance, we first evaluated differences in Hh signaling between EGFR-TKI-sensitive and -resistant NSCLC cells. The results showed that the Hh signaling pathway was aberrantly activated, accompanied by induction of an EMT phenotype and ABCG2 overexpression, in EGFR-TKI-resistant cells, whereas the Hh pathway was silenced in EGFR-TKI-sensitive cells. The difference in Hh signaling activity between EGFR-TKI-sensitive and -resistant cells suggested that aberrant activation of the Hh signaling pathway may contributes to EGFR-TKI resistance by inducing an EMT phenotype and ABCG2 upregulation. To confirm these findings, Hh signaling was upregulated using extrinsic SHH in the EGFR-TKI-sensitive PC9 cell line. Upregulation of Hh activity resulted in induction of EMT and elevation of ABCG2 expression. Hh signaling hyperactivity also resulted in EGFR-TKI tolerance in otherwise EGFR-TKI-sensitive cells. These results confirmed that aberrant activation of Hh signaling resulted in the development of EGFR-TKI resistance in NSCLC cells through induction of EMT and ABCG2 overexpression. Inhibition of Hh signaling by the Hh inhibitor SANT-1 enhanced E-cadherin expression and downregulated Snail and ABCG2 expression. This result demonstrated that blockade of Hh signaling can reverse the EMT phenotype and maybe reduce CSC abundance. A previous study indicated that restoring E-cadherin expression increased sensitivity to EGFR-TKIs *in vitro* and *in vivo*[38]. Based on these results, we had reason to believe that targeting the Hh signaling pathway could affect EGFR-TKI resistance in NSCLC cells. Thus, we next treated EGFR-TKI-resistant cells with gefitinib or SANT-1 alone or gefitinib plus SANT-1. The results indicated that compared with either gefitinib or SANT-1 alone, the combination of gefitinib plus SANT-1 significantly inhibited tumorigenesis and cell viability. Taken together, our findings suggest associations among Hh signaling, EMT, ABCG2 overexpression, and EGFR-TKI resistance in NSCLC cells for the first time.

Deregulation of Hh signaling contributes to tumorigenesis or accelerates tumor growth in an Hh ligand-independent or -dependent manner[43]. In most basal cell carcinomas (BCCs) and medulloblastomas, loss-of-function mutations in PTCH and gain-of-function mutations in SMO both lead to ligand-independent, mutation-driven activation of the Hh pathway[44–46]. This type of tumor can benefit from treatment with a single Hh inhibitor. In the development of several other types of cancer—such as prostate, breast, pancreatic, breast, and lung cancer—over- or ectopic expression of Hh ligands, which activate signaling in an autocrine or paracrine manner, lead to ligand-dependent Hh pathway activation[34, 47–51]. In this mechanism, the Hh signaling pathway plays a secondary role in tumor maintenance and growth. Thus, targeting the Hh pathway alone in these tumors is less likely to be successful unless combined with other appropriate chemotherapy or targeted therapy, or as a maintenance therapy[52]. Our data indicated that although SANT-1 reversed the EMT phenotype and decreased ABCG2 expression in EGFR-TKI-resistant cells, it had a minor inhibitory effect, especially in the A549 cell line. In contrast, the combination of SANT-1 with gefitinib effectively inhibited cellular proliferation and tumorigenesis. Patients with NSCLC may not benefit from single-agent Hh inhibitors. Combinations of Hh inhibitors and EGFR-TKIs may be an effective therapeutic strategy for primary and secondary EGFR-TKI-resistant NSCLCs. Compared with cytotoxic chemotherapy, targeted drugs possess the features of superior responses and fewer adverse effects, and thus have a broader treatment spectrum. Patients with ECOG performance status scores > 2 lose the opportunity to receive ‘traditional’ cytotoxic therapy. However, these patients can receive targeted therapy. Determining individual therapeutic schedules according to their molecular profiles is an increasing trend in cancer treatment. Our research indicated that an Hh inhibitor increased the sensitivity to EGFR-TKIs in EGFR-TKI- resistant cells that have an EMT phenotype and ABCG2 overexpression. NSCLCs harboring KRAS mutations may have even higher Hh signaling pathway activity. E-cadherin was strongly expressed in EGFR-TKI-sensitive NSCLC tissues. E-cadherin expression was negatively related to GLI1 expression. Previous studies have also reported that EMT is a determinant of EGFR-TKIs in NSCLCs *in vitro* and *in vivo*[35, 36]. Thus, E-cadherin expression may be a potential biomarker of suitability for combined application of Hh inhibitors and EGFR-TKIs in EGFR-TKI-resistant NSCLCs.

In conclusion, our study suggests that Hh signaling hyperactivity resulted in EGFR-TKI resistance due to EMT induction and ABCG2 upregulation. The combination of an Hh inhibitor and EGFR-TKIs may be an effective therapy for EGFR-TKI-resistant NSCLCs that harbor the EMT phenotype and ABCG2 overexpression. E-cadherin may be a useful biomarker to determine the suitability of application of Hh inhibitors in EGFR-TKI-resistant NSCLCs.

## Supporting Information

S1 Table The raw date of the proliferation of PC9 cells after treatment with indicated concentrations of Gefitinib with or without the pre exposure of extrinsic N-Shh (0.5ug/ml) for 24 hours analyzed by factorial analysis. (DOCX)

S2 Table The proliferation of PC9 cells after treatment with indicated concentrations of Gefitinib with or without the pre exposure of extrinsic N-Shh (0.5ug/ml) for 24 hours. (DOCX)

S3 Table The raw date of the proliferation effects after treatment with different concentration of Gefitinib single agent, SANT-1 single agent or the combination of Gefitinib and SANT-1 on A549 cells analyzed by factorial analysis. (DOCX)

S4 Table The effects of proliferation after treatment with different concentration of Gefitinib single agent, SANT-1 single agent or the combination of Gefitinib and SANT-1 on A549 cells. (DOCX)

S5 Table The raw date of the proliferation effects after treatment with different concentration of Gefitinib single agent, SANT-1 single agent or the combination of Gefitinib and SANT-1 on H1975 cells analyzed by factorial analysis. (DOCX)

S6 Table The effects of proliferation after treatment with different concentration of Gefitinib single agent, SANT-1 single agent or the combination of Gefitinib and SANT-1 on H1975 cells. (DOCX)
