# Supplementary material for: Blockade of Hedgehog Signaling Synergistically Increases Sensitivity to Epidermal Growth Factor Receptor Tyrosine Kinase Inhibitors in Non-Small-Cell Lung Cancer Cell Lines
Source: PLoS One. 2016 Mar 4;11(3):e0149370. doi: 10.1371/journal.pone.0149370 (PMC4778934; doi:10.1371/journal.pone.0149370)
Supplement: S1 Table — (DOCX) [file pone.0149370.s001.docx]

S1 Table. The raw date ofthe proliferation of PC9 cells after treatment with indicated concentrations of Gefitinib with or without the pre exposure of extrinsic N-Shh (0.5ug/ml) for 24 hoursanalyzed by factorial analysis.

**Tests of Between-Subjects Effects**

Dependent Variable: proliferation

| Source | Type III Sum of Squares | df | Mean Square | F | Sig. |
| --- | --- | --- | --- | --- | --- |
| Corrected Model | 2.901(a) | 7 | .414 | 94.659 | .000 |
| Intercept | 7.828 | 1 | 7.828 | 1787.805 | .000 |
| group | .385 | 1 | .385 | 87.962 | .000 |
| dose | 2.487 | 3 | .829 | 189.336 | .000 |
| group * dose | .029 | 3 | .010 | 2.215 | .126 |
| Error | .070 | 16 | .004 |  |  |
| Total | 10.799 | 24 |  |  |  |
| Corrected Total | 2.971 | 23 |  |  |  |

a R Squared = .976 (Adjusted R Squared = .966)
