# Supplementary material for: Blockade of Hedgehog Signaling Synergistically Increases Sensitivity to Epidermal Growth Factor Receptor Tyrosine Kinase Inhibitors in Non-Small-Cell Lung Cancer Cell Lines
Source: PLoS One. 2016 Mar 4;11(3):e0149370. doi: 10.1371/journal.pone.0149370 (PMC4778934; doi:10.1371/journal.pone.0149370)
Supplement: S2 Table — (DOCX) [file pone.0149370.s002.docx]

S2 Table.The proliferation of PC9 cells after treatment with indicated concentrations of Gefitinib with or without the pre exposure of extrinsic N-Shh (0.5ug/ml) for 24 hours.

| Method |  | Gefitinib | | | Total | *t/F*value | *P*value |
| --- | --- | --- | --- | --- | --- | --- | --- |
|  | 0nM | 20nM | 40nM | 80nM |  |  |  |
| G | 1.03±0.05 | 0.46±0.01 | 0.21±0.05 | 0.11±0.01 | 0.44±0.36 | 327.579 | <0.001 |
| S+G | 1.19±0.06 | 0.66±0.13 | 0.57±0.06 | 0.38±0.07 | 0.70±0.32 | 49.082 | <0.001 |
| Total | 1.09±0.11 | 0.56±0.14 | 0.39±0.20 | 0.24±0.15 | 0.57±0.36 | 189.336* | <0.001* |
| *t/F* value | -3.530 | -2.700 | -8.146 | -6.855 | 87.962* | 2.215# | 0.126# |
| *P* value | 0.024 | 0.054 | 0.001 | 0.002 | <0.001* |  |  |

G：Gifitinib；S：SHH；*main effect；#interaction effect
