# Supplementary material for: Blockade of Hedgehog Signaling Synergistically Increases Sensitivity to Epidermal Growth Factor Receptor Tyrosine Kinase Inhibitors in Non-Small-Cell Lung Cancer Cell Lines
Source: PLoS One. 2016 Mar 4;11(3):e0149370. doi: 10.1371/journal.pone.0149370 (PMC4778934; doi:10.1371/journal.pone.0149370)
Supplement: S3 Table — (DOCX) [file pone.0149370.s003.docx]

S3 Table.The raw date of the proliferation effectsafter treatment with different concentration ofGefitinib single agent, SANT-1 single agent or the combination of Gefitinib and SANT-1 on A549 cellsanalyzed by factorial analysis.

**Tests of Between-Subjects Effects**

Dependent Variable: proliferation

| Source | Type III Sum of Squares | df | Mean Square | F | Sig. |
| --- | --- | --- | --- | --- | --- |
| Corrected Model | .850(a) | 8 | .106 | 179.981 | .000 |
| Intercept | 12.983 | 1 | 12.983 | 21979.222 | .000 |
| group | .325 | 2 | .162 | 274.953 | .000 |
| group2 | .326 | 2 | .163 | 276.066 | .000 |
| group * group2 | .200 | 4 | .050 | 84.452 | .000 |
| Error | .011 | 18 | .001 |  |  |
| Total | 13.844 | 27 |  |  |  |
| Corrected Total | .861 | 26 |  |  |  |

a R Squared = .988 (Adjusted R Squared = .982)
