# Supplementary material for: Blockade of Hedgehog Signaling Synergistically Increases Sensitivity to Epidermal Growth Factor Receptor Tyrosine Kinase Inhibitors in Non-Small-Cell Lung Cancer Cell Lines
Source: PLoS One. 2016 Mar 4;11(3):e0149370. doi: 10.1371/journal.pone.0149370 (PMC4778934; doi:10.1371/journal.pone.0149370)
Supplement: S4 Table — (DOCX) [file pone.0149370.s004.docx]

S4 Table.The effects of proliferation after treatment with different concentration ofGefitinib single agent, SANT-1 single agent or the combination of Gefitinib and SANT-1 on A549 cells.

| Method | Concentration | | | Total | *t/F*value | *P*value |
| --- | --- | --- | --- | --- | --- | --- |
|  | G20nM+S20nM | G40nM+S40nM | G80nM+S40nM |  |  |  |
| G | 0.83±0.01 | 0.78±0.01 | 0.72±0.04 | 0.77±0.05 | 16.978 | 0.003 |
| S | 0.83±0.01 | 0.78±0.03 | 0.70±0.01 | 0.77±0.06 | 31.376 | 0.001 |
| S+G | 0.81±0.03 | 0.56±0.03 | 0.25±0.02 | 0.54±0.25 | 394.329 | <0.001 |
| Total | 0.82±0.02 | 0.71±0.11 | 0.55±0.23 | 0.40±0.20 | 276.066* | <0.001* |
| *t/F*value | 0.503 | 77.787 | 303.919 | 274.953* | 84.452# | <0.001# |
| *P* value | 0.628 | <0.001 | <0.001 | <0.001* |  |  |

G：Gifitinib；S：SHH；*main effect；#interaction effect
