# Supplementary material for: Blockade of Hedgehog Signaling Synergistically Increases Sensitivity to Epidermal Growth Factor Receptor Tyrosine Kinase Inhibitors in Non-Small-Cell Lung Cancer Cell Lines
Source: PLoS One. 2016 Mar 4;11(3):e0149370. doi: 10.1371/journal.pone.0149370 (PMC4778934; doi:10.1371/journal.pone.0149370)
Supplement: S5 Table — (DOCX) [file pone.0149370.s005.docx]

S5 Table.The raw date of the proliferation effectsafter treatment with different concentration ofGefitinib single agent, SANT-1 single agent or the combination of Gefitinib and SANT-1 on H1975 cells analyzed by factorial analysis.

**Tests of Between-Subjects Effects**

Dependent Variable: proliferation

| Source | Type III Sum of Squares | df | Mean Square | F | Sig. |
| --- | --- | --- | --- | --- | --- |
| Corrected Model | 1.401(a) | 8 | .175 | 314.600 | .000 |
| Intercept | 10.410 | 1 | 10.410 | 18694.582 | .000 |
| group | .711 | 2 | .356 | 638.881 | .000 |
| group2 | .539 | 2 | .269 | 483.786 | .000 |
| group * group2 | .151 | 4 | .038 | 67.867 | .000 |
| Error | .010 | 18 | .001 |  |  |
| Total | 11.821 | 27 |  |  |  |
| Corrected Total | 1.411 | 26 |  |  |  |

a R Squared = .993 (Adjusted R Squared = .990)
