# Supplementary material for: Blockade of Hedgehog Signaling Synergistically Increases Sensitivity to Epidermal Growth Factor Receptor Tyrosine Kinase Inhibitors in Non-Small-Cell Lung Cancer Cell Lines
Source: PLoS One. 2016 Mar 4;11(3):e0149370. doi: 10.1371/journal.pone.0149370 (PMC4778934; doi:10.1371/journal.pone.0149370)
Supplement: S6 Table — (DOCX) [file pone.0149370.s006.docx]

S6 Table. The effects of proliferation after treatment with different concentration ofGefitinib single agent, SANT-1 single agent or the combination of Gefitinib and SANT-1 on H1975 cells.

| Method | Concentration | | | Total | *t/F*value | *P* value |
| --- | --- | --- | --- | --- | --- | --- |
|  | G20nM+S20nM | G40nM+S40nM | G80nM+S40nM |  |  |  |
| G | 0.91±0.01 | 0.84±0.02 | 0.78±0.02 | 0.84±0.06 | 40.476 | <0.001 |
| S | 0.74±0.02 | 0.53±0.04 | 0.41±0.01 | 0.56±0.14 | 110.474 | <0.001 |
| S+G | 0.76±0.01 | 0.44±0.02 | 0.18±0.03 | 0.46±0.25 | 405.416 | <0.001 |
| 合计 | 0.80±0.08 | 0.60±0.18 | 0.45±0.26 | 0.62±0.23 | 483.786* | <0.001* |
| *t/F*value | 98.107 | 154.647 | 488.201 | 638.881* | 67.867# | <0.001# |
| *P*value | <0.001 | <0.001 | <0.001 | <0.001* |  |  |

G：Gifitinib；S：SHH；*main effect；#interaction effect
